# Supplementary material for: Marine subsidies mediate patterns in avian island biogeography
Source: Proc Biol Sci. 2020 Mar 11;287(1922):20200108. doi: 10.1098/rspb.2020.0108 (PMC7126081; doi:10.1098/rspb.2020.0108)
Supplement: Subsidized Avian Island Biogeography - Supplementary Material [file rspb20200108supp1.pdf]

Supplementary Information

Table 1S List of species included in analyses in taxonomic order, with relative abundance based on detections within 50 metres of an observer in point count surveys. Species were selected for analyses if they were known to breed in the Central Coast Region of British Columbia, based on Breeding Bird Atlas of BC (https://www.birdatlas.bc.ca).

Alpha Code	Common Name	Scientific Name	No. of Detections
SOGR	Sooty grouse	*Dendragapus fuliginosus*	11
ECDO	Eurasian collared-dove	*Streptopelia decaocto*	2
RUHU	Rufous hummingbird	*Selasphorus rufus*	230
HAWO	Hairy woodpecker	*Leuconotopicus villosus*	9
NOFL	Northern flicker	*Colaptes auratus*	8
OSFL	Olive-sided flycatcher	*Contopus cooperi*	1
PSFL	Pacific-slope flycatcher	*Empidonax difficilis*	525
STJA	Steller's jay	*Cyanocitta stelleri*	12
CORA	Common raven	*Corvus corax*	23
NOCR	Northwestern crow	*Corvus caurinus*	152
CBCH	Chestnut-backed chickadee	*Poecile rufescens*	172
BRCR	Brown creeper	*Certhia americana*	52
PAWR	Pacific wren	*Troglodytes pacificus*	543
GCKI	Golden-crowned kinglet	*Regulus satrapa*	215
RCKI	Ruby-crowned kinglet	*Regulus calendula*	2
VATH	Varied thrush	*Ixoreus naevius*	47
AMRO	American robin	*Turdus migratorius*	76
SWTH	Swainson's thrush	*Catharus ustulatus*	117
HETH	Hermit thrush	*Catharus guttatus*	183
CEDW	Cedar waxwing	*Bombycilla cedrorum*	4
OCWA	Orange-crowned warbler	*Oreothlypis celata*	571
YWAR	Yellow warbler	*Setophaga petechia*	2
YRWA	Yellow-rumped warbler	*Setophaga coronata*	26
TOWA	Townsend's warbler	*Setophaga townsendi*	234
WIWA	Wilson's warbler	*Cardellina pusilla*	23
SAVS	Savannah sparrow	*Passerculus sandwichensis*	1
WCSP	White-crowned sparrow	*Zonotrichia leucophrys*	1
FOSP	Fox sparrow	*Passerella iliaca*	128
SOSP	Song sparrow	*Melospiza melodia*	84
DEJU	Dark-eyed junco	*Junco hyemalis*	39
RECR	Red crossbill	*Loxia curvirostra*	109
PISI	Pine siskin	*Spinus pinus*	8

Methods

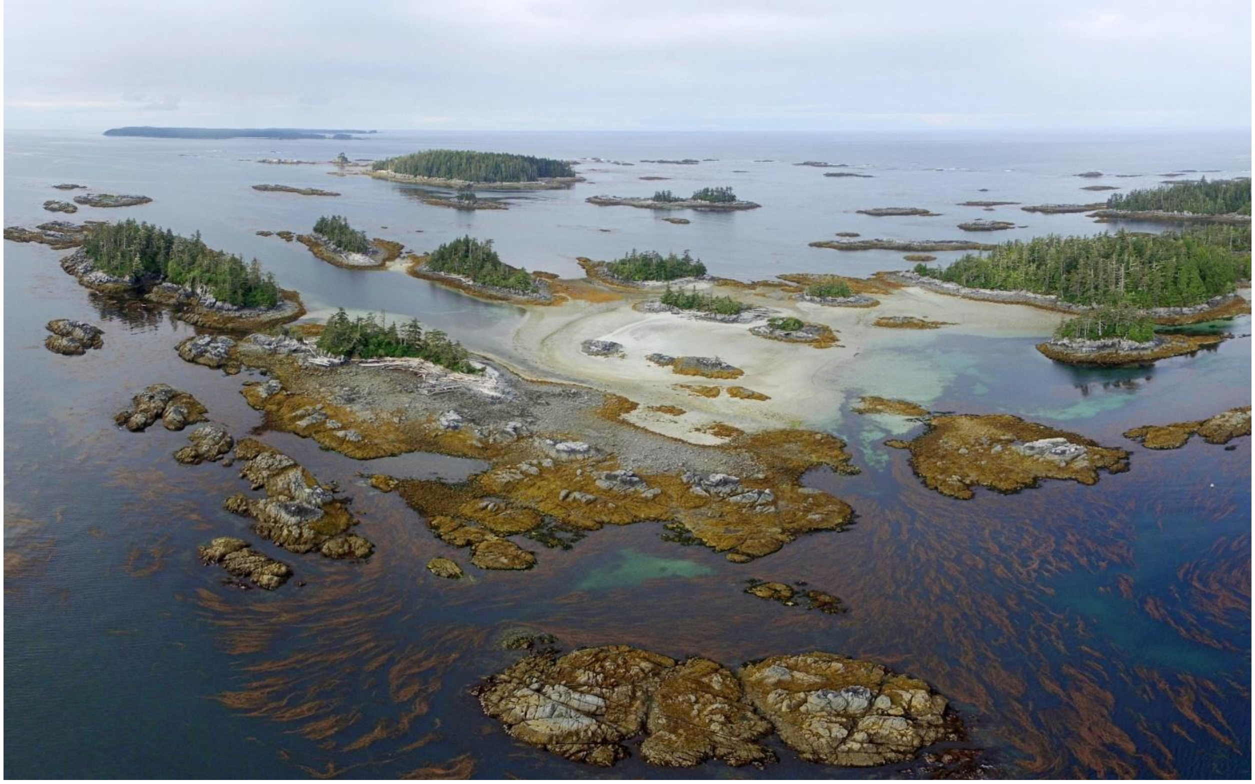

Figure 1S Temperate rainforest study islands, surrounded by a dynamic, productive matrix. Photo taken with a small Remotely Piloted Aerial System (sRPAS).

Island selection process:

Islands representative of the biogeographical and geomorphological variation in the region were chosen by using a two-step clustering method in SPSS statistical software (V23, IBM). This analysis considered 5 descriptors per island for the 1470 islands in the region: distance from mainland, area, exposure, normalized (size-independent) perimeter-to-area ratio, and percentage of area occupied by surrounding landmasses within 500 m of the island. For exposure, we used the British Columbia ShoreZone dataset which classifies a unit of shoreline with a given exposure classification from very exposed to very protected. These classifications are based on wave exposure categories derived from wind fetch distances.
The analysis used these variables to identify 5 clusters of island types (Table 2S). To facilitate sampling in a remote location, we selected 9 physical groupings of islands with 6-17 islands per group.

Table 2S Results of cluster analysis.

Cluster	# of Islands	Description
1	134	high exposure, close to mainland, few neighbouring islands
2	264	low exposure, close to mainland
3	432	high exposure, far from mainland, few neighbouring islands
4	426	low exposure, far from mainland, many neighbouring islands
5	197	low exposure, very close to mainland, many neighbouring islands, low P:A

Estimating isolation:

To create an isolation metric, we considered that the classical TIB prediction that species richness varies with distance to mainland does not apply to this system because birds are highly mobile, and numerous large islands serve as a functional "mainland" source population. Rather than using the classical “distance to mainland” metric, we predicted the minimum size of an island that acts as a functional "mainland", and then used distance to the nearest island of that size as a predictor for our models.

To do this, we used the ‘*nls*’ (non-linear least squares) function in R to fit a non-linear model to the unlogged, rarefied species richness data as a response to the unlogged area, using the Michaelis-Menten function for asymptotic data with a y-intercept of zero. The Michaelis-Menten formula is y = a*x / (x + b) (plus intercept which is 0), where a is the y value at highest rate of increase, and b is the x value at approximately 1/2 of the asymptote. We then extracted the area at which species richness reached 90% of the asymptotic value, and measured distance to the nearest island of that size. We compared these results with those obtained from extracting the area for 95% and 99% of the asymptote, and, although the size of island falling into these categories is widely variable, the number of species found on an island of 90% the asymptote is less than 1 fewer than on an island with area 99% of the asymptote, so we used the distance to the closest island able to hold 90% of the maximum number of observed species as a metric for isolation (Figure 2S).

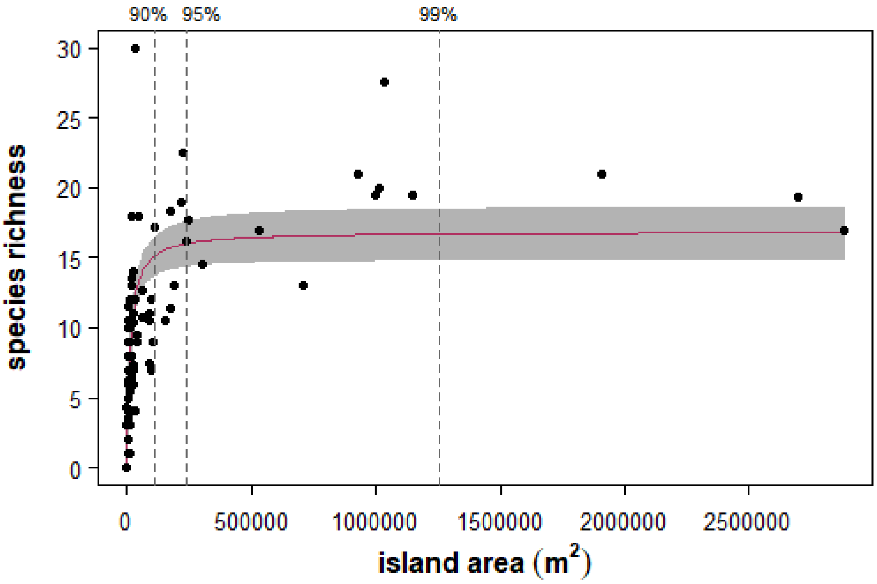

Figure 2S Species richness of terrestrial breeding birds plotted against island area. The red line represents the Michaelis-Menten function. Shaded area is the 95% confidence interval.

Analyses:

In a preliminary analysis, we evaluated the effects of isolation and habitat heterogeneity on bird species richness and population density. In the species richness analysis, there was no difference between the model including both area and isolation, and the model with area alone, even when

considering Burnham and Anderson's least stringent cutoff of a difference of <2 ΔAICc units [1]. Considering habitat heterogeneity in the model also proved to be uninformative (ie. the parameter did not improve model fit). All four models (area, area + isolation, area + habitat heterogeneity, and area + isolation + habitat heterogeneity) were better than the null model, which carried zero weight. In the population density model, adding habitat heterogeneity to the area model did not improve model quality, but both the area only model and area and habitat heterogeneity models were better than any models containing the isolation parameter.

Table 3S Initial suite of models to determine if isolation and habitat heterogeneity helped explain variation in species richness and improved model fit compared to island area alone. Even by the least stringent standards of <2 ΔAICc, these additional parameters are uninformative.

Model	*K*	*AICc*	*ΔAICc*	*AICc weight*	*Cumulative weight*	*Log-likelihood*
area + isolation	6	-34.61	0.00	0.46	0.46	23.80
area	5	-33.72	0.89	0.30	0.76	22.21
area + isolation + habitat heterogeneity	7	-32.31	2.30	0.15	0.91	23.83
area + habitat heterogeneity	6	-31.43	3.18	0.09	1.00	22.21
null	4	49.15	83.75	0.00	1.00	-20.34

Table 4S Initial suite of models to determine if isolation and habitat heterogeneity helped explain variation in population density and improved model fit compared to island area alone.

Model	*K*	*AICc*	*ΔAICc*	*AICc weight*	*Cumulative weight*	*Log-likelihood*
area	5	653.82	0.00	0.47	0.47	-321.56
area + habitat heterogeneity	6	654.89	1.07	0.28	0.75	-320.94
area + isolation	6	655.99	2.17	0.16	0.91	-321.50
area + isolation + habitat heterogeneity	7	657.22	3.40	0.09	1.00	-320.94
null	4	670.54	16.72	0.00	1.00	-331.04

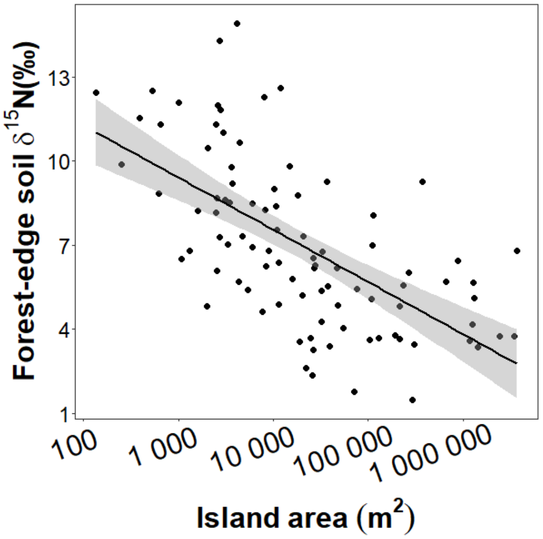

Figure 3S Correlation between marine input in the form of forest-edge soil δ15N and island area on 91 islands on the Central Coast of British Columbia, Canada. Correlation coefficient is -0.62, meaning that the variables are moderately correlated. Forest-edge soil $\delta^{15}N$ are averages from 4 plots representing the 4 cardinal directions at 0m from shore, so should not be affected by perimeter-to-area ratios.

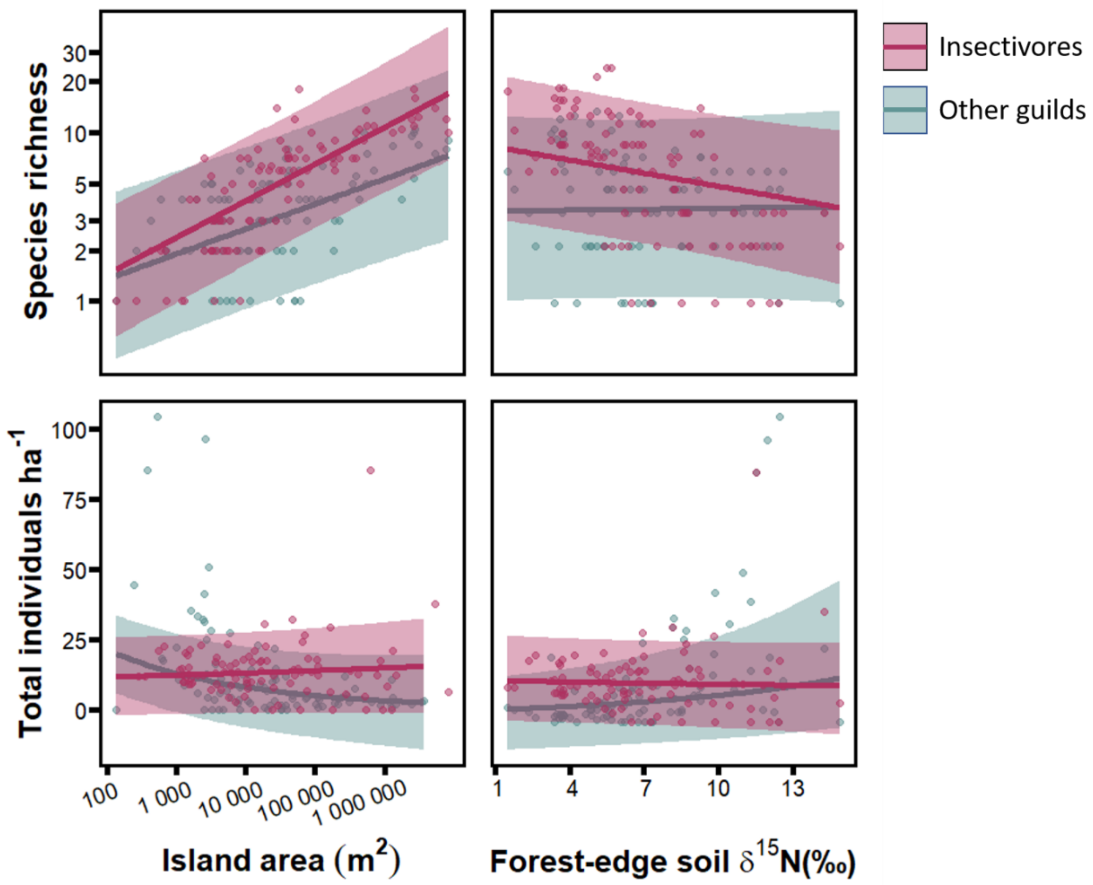

Figure 4S We fit separate global models for insectivore and “other guilds” species richness and total density to determine if any particular feeding guild was dropping out at higher levels of δ15N. We classified species based on the 5 diet categories described in the Elton Traits 1.0 database [2]. The “other” guild included “Omnivore”, “FruiNect”, “VertFishScav”, and “PlantSeed” feeding categories. We combined these other guilds because they were poorly represented overall in our study with just a few species in each. The majority (~2700 out of 3600) of our observations were of insectivorous birds. Area had a strong positive effect on species richness of both invertebrates and other guilds; however, the effect of δ^{15}N was only significant for insectivores. Neither area nor δ^{15}N had a significant effect on insectivore density, but area had a strong negative effect and δ^{15}N a positive albeit highly uncertain effect on the total bird density of other guilds. The interaction between area and δ^{15}N was also positive for the density of individuals in other guilds.

Relative Variable Importance (RVI):

To obtain meaningful RVIs, each parameter must occur in an equal number of models, so we considered all subsets of fixed effects and model-averaged across all outcomes to obtain coefficients and associated standard errors using the ‘*MuMIn’* package in R [3]. Testing all possible combinations of parameters is not recommended when trying to determine “significance” or trying to isolate a top model, but is an effective technique to determine RVIs [4, 5].

Literature cited

[1] Burnham KP, Anderson DR. 2002 Model Selection and Inference: A Practical Information-Theoretic Approach. 2nd Edition, Springer-Verlag, New York.
[2] Wilman H., Belmaker J., Simpson J., de la Rosa C., Rivadeneira MM, Jetz, W. 2014 EltonTraits 1.0: Species-level foraging attributes of the world's birds and mammals. *Ecology* **95**, 2027-2027. (doi:10.1890/13-1917.1)
[3] Barton K. 2019 MuMIn: Multi-model inference. R package version 1.43.6. **1**, 18.
[4] Arnold TW. 2010 Uninformative parameters and model selection using Akaike's Information Criterion. *J Wildl Manag* **74**, 1175-1178.
[5] Burnham KP, Anderson DR. 2004 Multimodel inference - understanding AIC and BIC in model selection. *Sociol. Methods Res.* **33**, 261-304. (doi:10.1177/0049124104268644).
